# Progress in Medicine

**Published:** 1898-12-24

**Authors:** 


### Progress in Medicine.

### DIPHTHERIA.

(*Continued from page* 198.)

**Diphtheria Therapy.** — Loeffler[28] has found the following mixture, distributed over a surface, kills the diphtheria germs in 20 seconds, viz.: Alcohol and turpentine equal parts, with 2 per cent. of carbolic acid. But Loeffler sought a preparation even more speedy in action, and as the result of extended investigation he felt assured that the best results are obtained from the following combination: Alcohol, 64 parts; benzole (benzene) or toluol (toluene) 36 parts; and that the activity of the solution is augmented by adding 4 per cent. of iron-chloride solution. In dealing with sensitive patients and children, Loeffler recommends the addition of 10 per cent menthol. He prepares his solution as follows: Menthol, 10 grammes; toluene, q.s. to make 36 cc.; then add—creolin 2 cc. iron-chloride solution (sesqui-chloride), 4 cc. alcohol absolute, q.s., to make 100 cc. In applying, the mucus is first wiped off, and then a pledget of wool soaked in the solution is pressed forcibly against the diphtheritic membrane for 10 seconds, and this application must then be immediately repeated. The applications are to be continued every three hours for four or five days, when all the local symptoms will probably have vanished. The solution does not appear to act so speedily on staplylococci and streptococci as on diphtheria bacilli.

**Serum Treatment.**—The second report of the Metropolitan Asylums Board[29] on 2,764 cases treated during 1896 shows that the mortality was 25·9 per cent. Taking them according to age, the percentage mortality is seen to diminish progressively from 32·2 among those under five years of age, to 4·2 at ages over twenty; while according to days of the disease, it rises from 5·2 in those who came under treatment on the first day to 31·7 in those admitted on the fifth day and after. The total mortality of all cases treated during 1896 (including those not treated with antitoxic serum) was 20·8, as compared with a mortality of 29 6 per cent. in all the cases treated in 1894, before the serum was used. Having regard only to those patients under five years of age, the mortality was found to be respectively 47·4 in 1894 and 30·2 in 1896. Very striking is the fact that nearly 60 per cent. of the tracheotomised children recovered in 1896, while in 1894 the mortality was more than double. As regards dosage, the average number of injections was 2·3; the average dose was 1,983 units per injection. With the strongest serum supplied 8,000 units can easily be given at one injection. "With such a serum the rule must be to give, in severe cases, 8,000 to 12,000 units when the patient is first seen, followed by another 2,000 to 8,000 units every twelve hours for the next 24 or 48 hours.

The conclusions arrived at by the whole of the Board, except Dr. Gayton, are as follows: (1) A great reduction in the mortality of cases brought under treatment on the first three days of illness; (2) the lowering of the combined general mortality to a point below that of any former year; (3) the still more remarkable reduction in the mortality of the laryngeal cases; (4) the uniform improvement in the results of tracheotomy at each separate hospital; (5) the beneficial effect produced on the clinical course of the disease.

The records of the University College cases reported by Martin and Hunt[30] are even more instructive. All the cases were admitted on clinical grounds as diphtheria, thus disposing of any fallacious comparison on the score of the mild cases which were formerly overlooked clinically being included solely on the bacteriological test. In all there were admitted in 1896 and 1897 a total of 185 cases, but of these seven must be excluded (viz., five which recovered without antitoxin because they were not considered to be true diphtheria, and two fatal cases in which antitoxin was only given by the mouth), leaving 178 cases treated with antitoxin. The effect on the mortality is best shown by comparing the mortality of the three antitoxin years with the preceding non-antitoxin years. The percentage mortality in the four non-antitoxin years was laryngeal cases, 70, 68, 78, 47; pharyngeal cases, 18·7, 14·3, 10·9, 30 0; total percentages, 43 5, 33·3, 37, 39. The same figures in the three antitoxin years successively were, laryngeal cases, 33·3, 40·0, 23·5; pharyngeal, 24 4, 10·0, 13·0; total percentages, 28·0, 17·7, 17·0. Again, they compare the mortality in the tracheotomy and intubation cases.

Mortality of Tracheotomy and Intubation Cases.

| Year. | Tracheotomy. | | Intubation. | | Total. | | |
|---|---|---|---|---|---|---|---|
| | Cases. | Died. | Cases. | Died. | Cases. | Died. | Mortality % |
| 1890 | 41 | 30 | 1 | 1 | 42 | 31 | 74 } 65·5 |
| 1891 | 17 | 15 | 17 | 9 | 34 | 24 | 70 |
| 1892 | 12 | 6 | 8 | 3 | 20 | 9 | 45 |
| 1893 | 39 | 30 | — | — | 39 | 30 | 77 |
| 1894 | 34 | 17 | — | — | 34 | 17 | 50 |
| 1895 | 25 | 5 | — | — | 25 | 5 | 20 } 26·4 |
| 1896 | 18 | 7 | — | — | 18 | 7 | 39 |
| 1897 | 29 | 7 | — | — | 29 | 7 | 24 |

And further evidence of the value of the early administration of antitoxin is shown by the table here quoted:—

Mortality according to Day of Disease.

| | Admitted from First to Fourth Day of Disease. | | | Admitted after Fourth Day of Disease. | | |
|---|---|---|---|---|---|---|
| | Cases. | Deaths | Percentage Mortality. | Cases. | Deaths | Percentage Mortality. |
| 1891 | 39 | 19 | 48·7 } 37 | 21 | 8 | 38·1 } 43 |
| 1892 | 31 | 11 | 35·5 | 24 | 9 | 37·5 |
| 1893 | 50 | 14 | 28·0 | 34 | 17 | 50·0 |
| 1894 | 36 | 14 | 38·8 | 23 | 10 | 43·0 |
| 1895 | 46 | 8 | 17·3 } 14 | 28 | 13 | 46·4 } 35 |
| 1896 | 66 | 9 | 13·6 | 24 | 7 | 29 0 |
| 1897 | 60 | 7 | 11·7 | 28 | 8 | 28·6 |

Another point of very great value is the very much better results obtained by employing larger doses of antitoxin than were formerly used. In the first series of mortality figures above cited it is shown that the total mortality of 1895, the first antitoxin year, was 28 per cent., which was much greater than that of 1896 and 1897, the second and third antitoxin years, in which it was 17·7 and 17 per cent. respectively. During 1895 the antitoxin used was obtained from various sources, its strength in normal units was not known, but it was certainly very weak, so weak that an insufficient dose (often under 1,000 normal units) was generally given. The only change in the treatment during the next two years was the administration of a more concentrated serum of known unit strength; and of this a much larger dose, averaging 7,200 units in 1896 and 7,800 units in 1897, was employed. In the later year six-sevenths of the cases received over 6,000 normal units, and it is now the practice of the hospital to give each patient on admission a dose of not less than 6,000 normal units. One of the most important and instructive results shown by the author's cases is this great saving of life which has been effected by the increase of the dose of serum. In 1897 the number of units given was between 4,000 and 12,000, the average being 7,000 to 8,000.

Local treatment was employed in almost all the cases. In the majority swabs of chloride of zinc (grs. x to ℥j) were employed; in others sprays of perchloride of mercury or douches of chlorine water. All cases had steam sprays of bicarbonate of soda at two-hourly intervals. The authors remark that when we consider the very frequent association of pus and septic micro-organisms with the diphtheria bacillus in the throat, it is clear that we must not rely on antitoxin alone, but must also use local antiseptics in order to obtain the best results. They also demonstrate the beneficial effect of the antitoxin treatment in causing a decline in the temperature earlier than in the pre-antitoxin days.

[28] Deut. Med. Leit. Laryngoscope, Sept., 1898. [29] Brit Med. Jour., May 29, 1897. [30] Loc. cit.

